# Expression of Extracellular Matrix Components Is Disrupted in the Immature and Adult Estrogen Receptor β-Null Mouse Ovary

**DOI:** 10.1371/journal.pone.0029937

**Published:** 2012-01-10

**Authors:** Alexandra Zalewski, Erin L. Cecchini, Bonnie J. Deroo

**Affiliations:** 1 Department of Biochemistry, Schulich School of Medicine & Dentistry, The University of Western Ontario, London, Ontario, Canada; 2 Children's Health Research Institute, London, Ontario, Canada; Institut de Génomique Fonctionnelle de Lyon, France

## Abstract

Within the ovary, Estrogen Receptor β (ERβ) is localized to the granulosa cells of growing follicles. 17β-estradiol (E2) acting via ERβ augments the actions of follicle stimulating hormone in granulosa cells, leading to granulosa cell differentiation and formation of a preovulatory follicle. Adult ERβ-null females are subfertile and possess ovaries with reduced numbers of growing follicles and corpora lutea. Because the majority of E2 production by granulosa cells occurs once puberty is reached, a role for ERβ in the ovary prior to puberty has not been well examined. We now provide evidence that lack of ERβ disrupts gene expression as early as post-natal day (PND) 13, and in particular, we identify a number of genes of the extracellular matrix (ECM) that are significantly higher in ERβ-null follicles than in wildtype (WT) follicles. Considerable changes occur to the ECM occur during normal folliculogenesis to allow for the dramatic growth, cellular differentiation, and reorganization of the follicle from the primary to preovulatory stage. Using quantitative PCR and immunofluorescence, we now show that several ECM genes are aberrantly overexpressed in ERβ-null follicles. We find that Collagen11a1, a protein highly expressed in cartilage, is significantly higher in ERβ-null follicles than WT follicles as early as PND 13, and this heightened expression continues through PND 23–29 into adulthood. Similarly, Nidogen 2, a highly conserved basement membrane glycoprotein, is elevated in ERβ-null follicles at PND 13 into adulthood, and is elevated specifically in the ERβ-null focimatrix, a basal lamina-like matrix located between granulosa cells. Focimatrix laminin and Collagen IV expression were also higher in ERβ-null ovaries than in WT ovaries at various ages. Our findings suggest two novel observations: a) that ERβ regulates granulosa cell gene expression ovary prior to puberty, and b) that ERβ regulates expression of ECM components in the mouse ovary.

## Introduction

It is well established that estrogens play a critical role in the ovary during folliculogenesis. 17β-estradiol (E2) synergizes with follicle stimulating hormone (FSH) to induce granulosa cell differentiation and the formation of a healthy preovulatory follicle capable of ovulation in response to luteinizing hormone (LH) [1]. E2 acts directly on granulosa cells [2], [3] via its receptor, ERβ [4], [5], which is the predominant ER form expressed in granulosa cells of both humans and mice.

E2 and ERβ are essential for folliculogenesis in mice. Adult ERβ-null females are sub-fertile or infertile [6], [7], [8], possess ovaries with reduced numbers of growing follicles and corpora lutea and, due to infrequent ovulation, have litters one-third the size of wildtype (WT) females or are completely sterile [6], [7], [8]. There is almost a complete lack of antral follicles in the prepubertal ERβ-null ovary [7]. Furthermore, ERβ-null granulosa cells isolated from post-natal day (PND) 23 mice have an attenuated response to FSH, resulting in reduced cAMP accumulation [5], and poorly differentiated granulosa cells [4]. This lack of differentiation results in attenuated follicular production of cAMP in response to LH [9], and reduced ovulation. Therefore, an important role for E2 and ERβ in the response to FSH in the ovaries of adult mice has been firmly established; however, a role for ERβ in the postnatal/immature ovary has not been explored. Lack of ERβ in the immature ovary might contribute to the impaired FSH response observed in ERβ-null granulosa cells.

Several lines of evidence indicate that both E2 and ERβ are not only present in the ovaries of immature rodents, but that E2 acting through ERβ regulates folliculogenesis at this time. E2 has been detected in neonatal circulation in the rat [10]. In addition, androstenedione (which can be converted to E2) is detectable at PND 7 in the mouse, and increases by PND 15 [11]. ERβ protein is present [12], [13], [14] and functional [13] in primary follicles in PND 4 mouse ovaries, consistent with earlier data indicating that ERβ mRNA is detectable in the mouse ovary as early as PND1 [14] or PND 4 [13], [14], and increases dramatically by PND 12 in the mouse [14] and rat [15]. Thus, both E2 and ERβ protein are simultaneously present in mice as early as PND 4, and increase around PND 12–15, when the ovary contains primordial and primary follicles, as well as secondary follicles with 2–3 layers of granulosa cells [16].

Evidence also suggests that E2, acting through ERβ, may regulate development of primordial and primary follicles. First, adult female *Cyp19a1*-null mice (which lack the enzyme Cyp19a1, also known as aromatase, which converts testosterone to 17β-estradiol in granulosa cells) have reduced numbers of primordial and primary follicles compared with WT mice [17], suggesting that production of E2 is required for optimal primordial and primary follicle development. Second, adult female ERβ-null mice have elevated numbers of primordial follicles, but reduced numbers of primary follicles [18]. Third, treatment of PND 20 mice with the ERβ-selective agonist 8β-VE_2_ significantly increases the number of primary follicles, while the ERα-selective agonist, 16α-LE_2_ did not [19]. These data suggest that E2 acting through ERβ may regulate the formation of primordial and/or primary follicles in young mice.

Based on these data, we hypothesized that disrupted gene expression would be observed in the ovaries of immature ERβ-null mice. The ERβ-null ovarian phenotype has been described almost exclusively in adult or gonadotropin-treated PND 23–29 mice; however, few studies have examined ERβ-null immature ovaries. Therefore, we examined the expression of a subset of genes (originally identified by microarray analysis [5] of granulosa cells isolated from PND 23–29 ERβ-null mice) in ERβ-null ovaries as early as PND 13. Specifically, we focussed our analysis on proteins of the extracellular matrix because functional analysis of the microarray data revealed the novel observation that many ECM genes were dysregulated in ERβ-null granulosa cells, suggesting a novel phenotype in ERβ-null ovaries not previously reported.

It is well established that dramatic changes in the ECM occur throughout folliculogenesis to allow for the dramatic growth of the follicle from the primary to preovulatory stage [20], [21], [22], [23], [24], [25], [26], [27]; the ECM regulates follicular cell morphology, aggregation, communication, differentiation, steroidogenesis, survival, and proliferation [27]. Two main follicular ECMs are the basal lamina and the “focimatrix,” a basal lamina-like matrix located between granulosa cells, and granulosa cells are thought to produce many of these ECM components [22], [23]. In this study, we chose to further characterize the expression and ovarian localization of two ECM proteins whose expression was higher in ERβ-null granulosa cells than in WT cells, suggesting that ERβ may repress their expression: Collagen 11a1 (*Col11a1*) and Nidogen 2 (*Nid2*). We characterize COL11A1 and NID2 localization and mRNA levels in the ovaries of immature mice at PND 13 and PND 23–29, as well as in adult mice. We also investigate several other ECM proteins (COL4, NID1, and Laminin) which were not identified as differentially regulated in the original microarray, but whose ovarian expression has been previously characterized in the mouse [20], [24]. Surprisingly, many of these ECM proteins are elevated as well in the ERβ-null ovary, suggesting a general disruption of ECM composition, and a potential role for this disruption in the reduced fertility observed in ERβ-null mice.

Therefore, the overall aim of our study was to demonstrate that gene expression is dysregulated in the immature ERβ-null ovary, and in particular, that extracellular matrix (ECM) gene expression is dysregulated. We now report for the first time that the expression of several ECM genes is dysregulated in the ERβ-null ovary as early as PND 13, and that this dysregulation is maintained within the adult ERβ-null ovary, resulting in altered expression of ECM components compared to WT mice. Taken together, our data identify two novel findings: a) that ERβ regulates gene expression in the mouse ovary much earlier than previously thought, and b) that ERβ plays a role in the regulation of ECM composition in the immature and adult mouse ovary.

## Results

Our previous microarray studies (Gene Expression Omnibus accession number GSE11585) [5] comparing the gene expression profiles of granulosa cells isolated from gonadotropin-treated immature (PND 23–29) ERβ-het (ERβ^+/−^) and ERβ-null (ERβ^−/−^) mice indicated that the expression of numerous extracellular (ECM) proteins was dysregulated in ERβ-null granulosa cells compared to ERβ-het cells (Table S1). From this set of ECM proteins (Table S1), we chose to further characterize the expression and ovarian localization of two proteins whose expression was higher in ERβ-null granulosa cells than in ERβ-het cells: Collagen 11a1 (*Col11a1*) and Nidogen 2 (*Nid2*). We focussed on these two proteins because they met the following four criteria: 1) follow-up studies confirming the microarray data indicated that both genes were dysregulated in granulosa cells isolated from *untreated* ERβ-null PND 23–29 mice, suggesting an earlier role for ERβ in ovarian development than previously thought, 2) the higher levels of expression in ERβ-null granulosa cells compared to ERβ-het cells suggested a novel inhibitory role for ERβ in the regulation of their expression (rather than an activational role), 3) there is previously-reported evidence for regulation of *Col11a1* (Gene Expression Omnibus dataset GDS884) and *Nid2* expression by 17β-estradiol [28], [29], and 4) the fold difference between ERβ-het and ERβ-null granulosa cells was greater than two, our predetermined cut-off value for further analysis. In addition, to our knowledge, expression of Collagen 11a1 had not been previously reported in the ovary, suggesting that its aberrantly high expression in ERβ-null granulosa cells may contribute to the disrupted folliculogenesis observed in ERβ-null mice. Note that untreated mice were used for all studies, ie. mice were not primed with gonadotropins or estradiol.

Therefore, we wanted to investigate COL11A1 and NID2 expression and localization at PND 13 and PND 23–29 to determine when dysregulated gene expression could first be detected in the ERβ-null ovary. We also investigated these genes in adult ovaries to determine if the dysregulation observed in immature mice was maintained in the adult ovary.

### COLLAGEN 11A1

**Figure 1:**
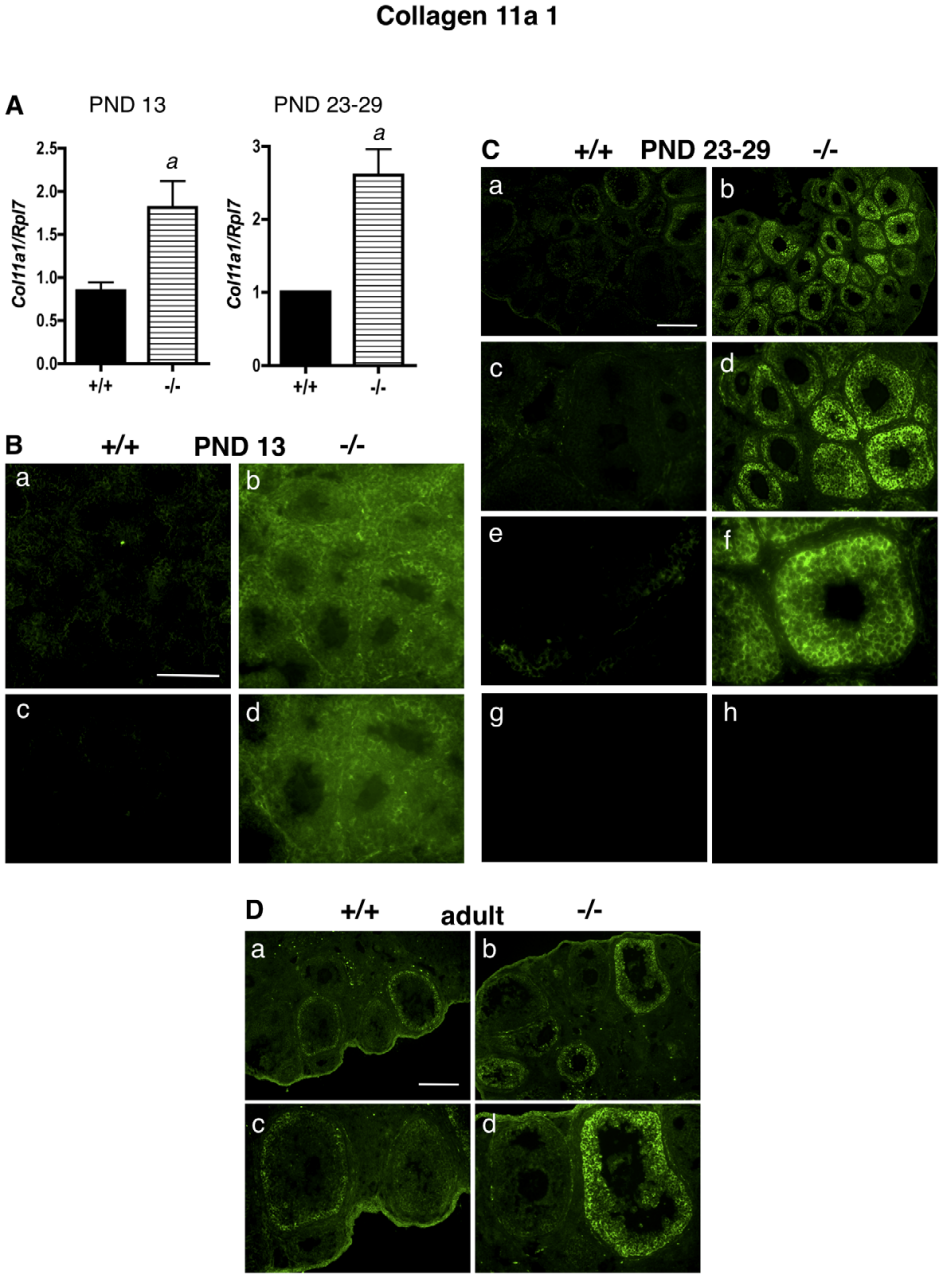
Collagen 11a1 mRNA and protein levels are higher in granulosa cells and ovaries of ERβ-null mice than in wildtype mice. A. Granulosa cells were isolated and pooled from ovaries of untreated PND 13 or PND 23–29 wildtype (+/+) or ERβ-null (−/−) mice, and the levels of *Col11a1* mRNA were determined by quantitative RT-PCR compared to an *Rpl7* control (± SEM of three independent experiments). Wildtype and ERβ-null average mRNA levels were compared using an unpaired two-tailed Student's t-test. a: p<0.05. B–D. Immunofluorescence with an anti-COL11A1 antibody was used to detect COL11A1 localization and expression in ovaries isolated from wildtype (+/+) and ERβ-null (−/−) mice at (B) PND 13 (a–d), (C) PND 23–29 (a–f; negative controls with secondary antibody only are shown in g and h), and (D) PND 60 (adult). Various magnifications are shown. (B) Scale bar = 100 µM for a–b, and 50 µM for c–d. (C) Scale bar = 200 µM for a–b and g–h, 100 µM for c–d, and 50 µM for e–f; (D) Scale bar = 200 µM for a–b, 100 µM for c–d.

At PND 13, *Col11a1* mRNA levels were approximately two-fold higher in ERβ-null whole ovaries than in WT ovaries, as determined by quantitative RT-PCR (qPCR) (Figure 1A). Similarly, *Col11a1* mRNA levels were 2.5-fold higher in granulosa cells isolated from PND 23–29 ERβ-null mice than in WT granulosa cells isolated from age-matched mice (Figure 1A). We then wanted to determine, using immunofluorescence: a) if these increases in *Col11a1* mRNA levels correlated with increases in protein expression, and b) the localization of COL11a1 within the immature and adult ovaries of WT and ERβ-null mice. At PND 13, when the mouse ovary contains many preantral follicles with 2–3 rows of granulosa cells surrounded by a basal lamina, in addition to primary and primordial follicles [16], ERβ-null ovaries expressed higher levels of COL11A1 than WT mice of the same age (Figure 1B), and COL11A1 appeared to be localized to the cytoplasm and extracellular region of granulosa cells. AT PND 23–29 (Figure 1C) COL11A1 was almost undetectable in WT PND 23–29 ovaries. However, COL11A1 was dramatically elevated in the follicles of ERβ-null mice (Figure 1C). COL11A1 protein was localized primarily to the cytoplasm of granulosa cells (Figure 1C, panel f). Similar localization in the follicle was observed for calnexin, which localizes to the endoplasmic reticulum and is frequently used as a cytoplasmic marker (Figure S1). COL11A1 expression was primarily observed in preantral follicles (both small and large), which predominate in the immature ERβ-null ovary. Only very weak COL11A1 staining was observed in the thecal layer or ovarian interstitium. In adult mice, as observed in the immature mice, COL11A1 expression was again higher in the granulosa cell cytoplasm in ERβ-null ovaries than in WT ovaries (Figure 1D).

### NIDOGEN 2

**Figure 2:**
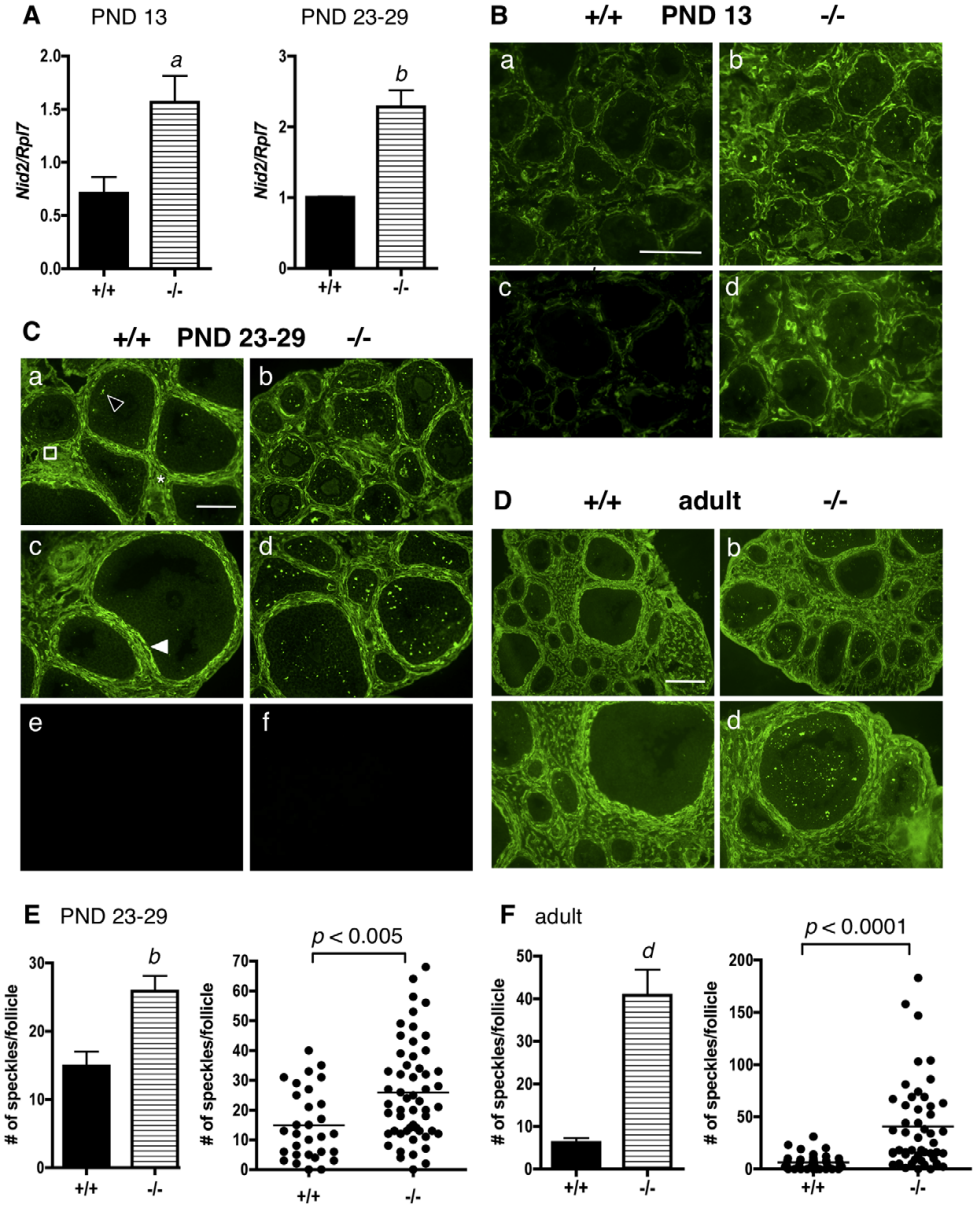
Nidogen 2 mRNA and protein levels are higher in granulosa cells and ovaries of ERβ-null mice than in wildtype mice. A. Granulosa cells were isolated and pooled from ovaries of untreated PND13 or PND 23–29 wildtype (+/+) or ERβ-null (−/−) mice, and the levels of *Nid2* mRNA were determined by quantitative RT-PCR compared to an *Rpl7* control (± SEM of three independent experiments). Wildtype and ERβ-null average mRNA levels were compared using an unpaired two-tailed Student's t-test. a: *p*<0.05; b: *p*<0.01. B–D. Immunofluorescence with an anti-NID2 antibody was used to detect NID2 localization and expression in ovaries isolated from wildtype (+/+) and ERβ-null mice (−/−) at (B) PND 13 (a–d), (C) PND 23–29 (a–d; negative controls with secondary antibody only are shown in e and f), and (D) PND 60 (adult). Various magnifications are shown at each age. (B) Scale bar = 100 µM for a–b, and 50 µM for c–d. (C) Two different sections from each genotype are shown (same magnification for both sections). Scale bar = 100 µM for a–f; (D) Scale bar = 200 µM for a–b, 100 µM for c–d. NID2 is localized to the follicular basal lamina (white filled arrowhead), focimatrix (open arrowhead), thecal matrix (asterix), and endothelial basal lamina of stromal blood vessels (square). (E, F) Focimatrix speckles in the PND 23–29 and adult sections were counted per follicle, and the difference between genotypes analyzed by a two-tailed, un-paired Student's t-test (± SEM, left panel) and by Receiver Operating Characteristic analysis (right panel). Each dot in the scatter plot (right panel) represents one follicle. b: *p*<0.01; d: *p*<0.0001.

At PND 13, *Nid2* mRNA levels were approximately 1.5-fold higher in ERβ-null whole ovaries than in WT ovaries, as determined by qPCR (Figure 2A). Similarly, *Nid2* mRNA levels were approximately 2.3-fold higher in granulosa cells isolated from PND 23–29 ERβ-null mice than in WT granulosa cells isolated from age-matched mice (Figure 2A). With respect to localization of NID2 within the ovary as determined by immunofluorescence, while COL11A1 localized almost exclusively to the cytoplasm of granulosa cells (Figure 1C), NID2 was localized to the follicular basal lamina, thecal matrix, sub-endothelial basal lamina of stromal blood vessels, and in a punctate pattern as “speckles” or “plaques” between granulosa cells (known as focimatrix) (Figure 2C) of PND 23–29 WT mice, as previously reported [24]. The focimatrix (**foc**al **i**ntra-epithelial **matrix**; a term coined by Irving-Rodgers et al. [30]), is a specialized ECM composed of basal-lamina like material that exists as plaques or aggregated deposits between granulosa cells, but does not surround the cells as a true basal lamina. Focimatrix is found in the ovaries of many species. In the mouse, primary focimatrix components include collagen, type IV α1 and α2, laminin α1, β1 and γ1, nidogens 1 and 2, perlecan, and collagen type XVIII [24]. Granulosa cells express mRNA encoding many focimatrix proteins [23], [31], and granulosa cells are thought to be the source of focimatrix protein production [25]. In our study, NID2 localization was similar in both WT and ERβ-null ovaries (Figure 2B and 2C) at PND 13 and PND 23–29. However, as predicted by the mRNA levels (Figure 2A), NID2 expression was higher in the follicles of ERβ-null mice (Figures 2B and 2C) than in WT mice at both ages. However, this increase was only observed in the focimatrix of ERβ-null ovaries; NID2 levels in the follicular basal lamina, thecal matrix, and sub-endothelial basal lamina of stromal blood vessels were similar in both genotypes. These differences in focimatrix NID2 expression between WT and ERβ-null follicles were quantified in PND 23–29 ovaries by counting the number of focimatrix speckles per follicle, and the difference tested for statistical significance (Figure 2E). A statistically significant difference in the number of focimatrix speckles per follicle was observed between WT and ERβ-null follicles (Figure 2E) on average, as determined by Student's t-test (Figure 2E, left panel). In addition, a statistically significant difference was also detected using the more stringent criteria of Receiver Operating Characteristic (ROC) analysis (Figure 2E, right panel), an analysis that tests for differences over the entirety of both distributions. At PND 13, ERβ-null ovaries again expressed higher levels of NID2 protein than WT mice of the same age (Figure 2B). Interestingly, NID2 expression appeared higher throughout the ovary of ERβ-null mice at this stage: in the focimatrix, in the follicular basal lamina and in thecal matrix. (Focimatrix speckles were not counted due to difficulty of accurate counts resulting from the irregularity of follicle shapes and sizes at this stage). Expression of NID2 was strikingly and significantly higher (Figure 2D and 2F) in adult ERβ-null focimatrix than in WT focimatrix, while expression of NID2 in other follicular compartments was similar in both genotypes, as observed in younger mice (Figures 2B and 2C).

To show that this difference in expression between ERβ-null and WT granulosa cells was specific to *Nid2* and *Col11a1*, but not to all ECM genes, we also investigated the expression of Nidogen 1 *(Nid1)*, Collagen, type IV (*Col4a1)*, and Laminin (*Lama1*). We chose the *Nid1*, *Col4a1*, and *Lama1* genes because their expression and localization has been previously characterized in the mouse ovary [20], [24], [32], and because neither gene had been detected as differentially expressed between WT and ERβ-null granulosa cells by our previously-conducted microarray (Table S1). Nidogen 1 is structurally similar to Nidogen 2 and shares overlapping expression patterns during development and in many adult tissues [33], [34], and both Collagen, type IV and Laminin are ubiquitous ECM proteins found in many tissues, including the ovary.

### NIDOGEN 1

**Figure 3:**
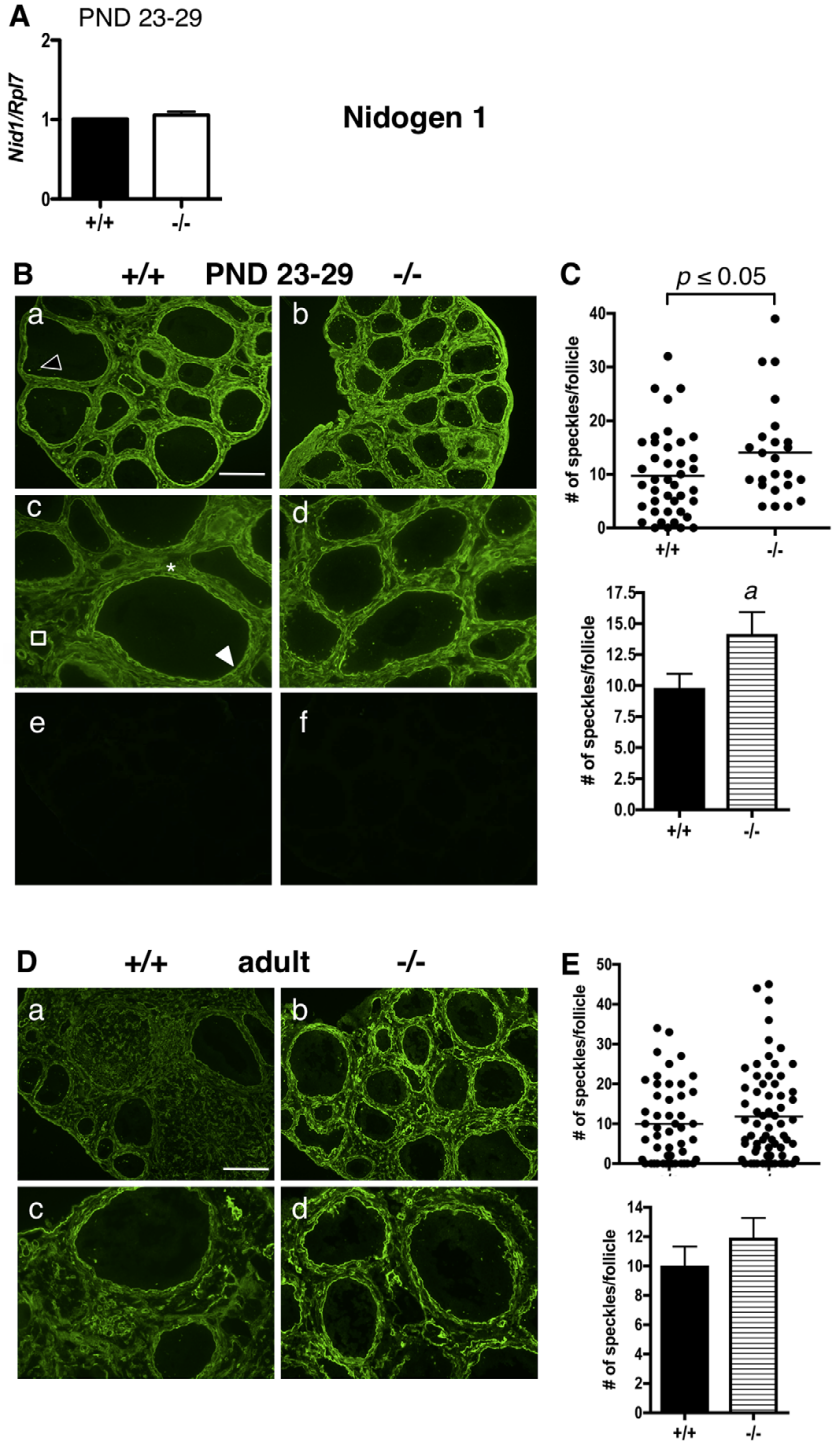
Nidogen 1 expression and localization in immature and adult ERβ-null and wildtype mouse ovaries. A. Granulosa cells were isolated and pooled from ovaries of untreated PND 23–29 wildtype (+/+) or ERβ-null (−/−) mice, and the levels of *Nid1* mRNA were determined by quantitative RT-PCR compared to an *Rpl7* control (± SEM of three independent experiments). B. Immunofluorescence with anti-NID1 antibodies was used to detect NID1 localization and expression in ovaries isolated from wildtype (+/+) and ERβ-null (−/−) mice at PND 23–29 (a–d; negative controls with secondary antibody only are shown in e and f). NID1 was localized to the follicular basal lamina (white filled arrowhead), focimatrix (open arrowhead), thecal matrix (asterix), and endothelial basal lamina of stromal blood vessels (square). NID1 focimatrix expression is slightly higher in ERβ-null ovaries than in wildtype ovaries at PND 23–29. Scale bar = 200 µM for a–b and e–f, 100 µM for c–d. C. A significant increase in NID1 expression within the focimatrix of ERβ-null ovaries compared to wildtype ovaries was observed at PND 23–29, as determined by the number of focimatrix “speckles” counted per follicle. Differences in the number of speckles/follicle between genotypes were analyzed by Receiver Operating Characteristic analysis (top panel) and a two-tailed, un-paired Student's t-test (± SEM, bottom panel). Each dot in the scatter plot (top panel) represents one follicle. a: *p*<0.05. D. NID1 expression in adult ERβ-null and wildtype mouse ovaries. Immunofluorescence with anti-NID1 antibodies was used to detect NID1 localization and expression in ovaries isolated from adult wildtype (+/+) and ERβ-null (−/−) mice. Two magnifications are shown. Scale bar = 200 µM for a–b, 100 µM for c–d. E. Expression of NID1 in the adult focimatrix was quantified by counting the number of focimatrix speckles/follicle, and these values were compared between genotypes by Receiver Operating Characteristic analysis (E, top panel) and a two-tailed, un-paired Student's t-test (± SEM, E bottom panel) in each case. Each dot in the scatter plot (E, top panel) represents one follicle. No statistically significant difference in NID1 focimatrix was observed between genotypes in the adult ovary.

*Nid1* mRNA levels were similar in both ERβ-null and WT granulosa cells at PND 23–29 (Figure 3A). Similar to NID2, NID1 localized to the follicular basal lamina, thecal matrix, focimatrix, and basal lamina of stromal blood vessels (Figure 3B) of PND 23–29 WT mice as previously reported [24]. No differences in NID1 expression levels were observed between genotypes in the follicular basal lamina, thecal matrix, or basal lamina of stromal blood vessels. Unexpectedly, NID1 expression in the focimatrix was slightly higher in ERβ-null follicles than in WT follicles (Figure 3B), and this increase was statistically significant (Figure 3C). No significant differences were observed in NID1 expression (Figures 3D and E) between adult wildtype and ERβ-null mice in the focimatrix, although the overall signal in the basal lamina and stroma appeared higher in ERβ-null ovaries than in WT ovaries.

### COLLAGEN IV

**Figure 4:**
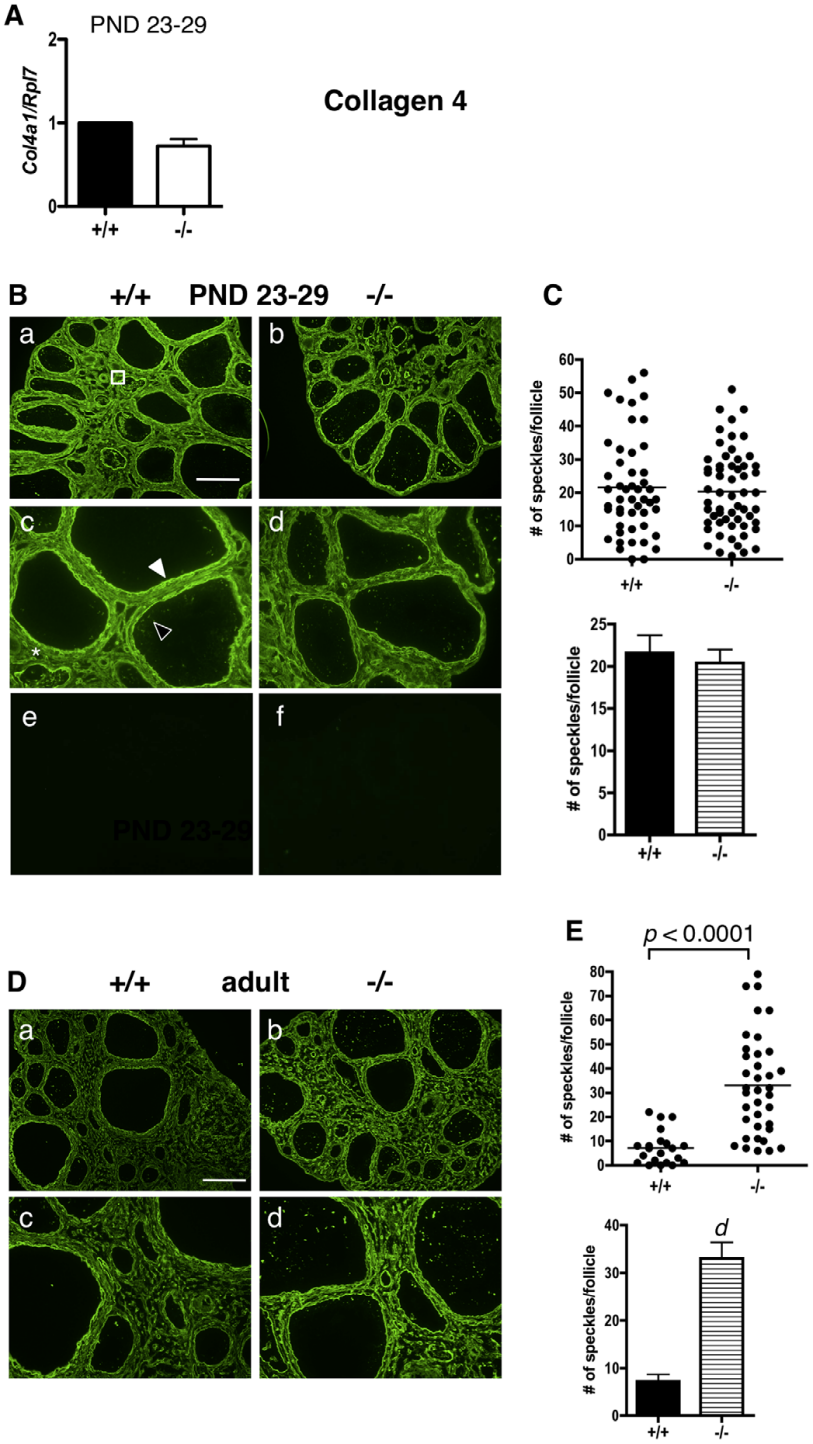
Collagen IV expression and localization in immature and adult ERβ-null and wildtype mouse ovaries. A. Granulosa cells were isolated and pooled from ovaries of untreated PND 23–29 wildtype (+/+) or ERβ-null (−/−) mice, and the levels of *Col4* mRNA were determined by quantitative RT-PCR compared to an *Rpl7* control (± SEM of three independent experiments). B. Immunofluorescence with an anti-COL4 antibody was used to detect COL4 localization and expression in ovaries isolated from wildtype (+/+) and ERβ-null (−/−) mice at PND 23–29 (a–d; negative controls with secondary antibody only are shown in e and f). COL4 was localized to the follicular basal lamina (white filled arrowhead), focimatrix (open arrowhead), thecal matrix (asterix), and endothelial basal lamina of stromal blood vessels (square). Scale bar = 200 µM for a–b and e–f, 100 µM for c–d. C. No significant differences in COL4 expression within the focimatrix were observed, as determined by the number of focimatrix “speckles” counted per follicle analyzed by Receiver Operating Characteristic analysis (top panel) and a two-tailed, un-paired Student's t-test (± SEM, bottom panel). Each dot in the scatter plot (top panel) represents one follicle. D. COL4 expression in adult ERβ-null and wildtype mouse ovaries. Immunofluorescence with anti-COL4 antibodies was used to detect COL4 localization and expression in ovaries isolated from adult wildtype (+/+) and ERβ-null (−/−) mice. Two magnifications are shown. Scale bar = 200 µM for a–b, 100 µM for c–d. E. Expression of COL4 in the focimatrix was quantified by counting the number of focimatrix speckles/follicle, and these values were compared between genotypes by Receiver Operating Characteristic analysis (top panel) and a two-tailed, un-paired Student's t-test (± SEM, bottom panel). Each dot in the scatter plot (bottom panel) represents one follicle. d: *p*<0.0001.

*Col4* mRNA levels were similar in both ERβ-null and WT granulosa cells at PND 23–29 (Figure 4A). Similarly, COL4 protein levels were the same in WT and ERβ-null mice (Figure 4B). Interestingly, the localization of COL4 and COL11A1 was not the same within the WT or ERβ-null ovary. While COL11A1 localized almost exclusively to the cytoplasm of granulosa cells (Figure 1C), COL4 staining was observed in the follicular basal lamina, the focimatrix, the thecal matrix, and in the stromal sub-endothelial basal lamina of blood vessels (Figure 4B), as previously reported for WT mice [20], [24], [32]. Similar COL4 localization and staining intensity was observed in WT and ERβ-null PND 23–29 ovaries (Figure 4A). Focimatrix COL4 expression was quantified by counting the number of focimatrix speckles per follicle (Figure 4C). As predicted by the mRNA levels (Figure 4A), no statistically significant differences in the number of focimatrix speckles per follicle were observed between WT and ERβ-null follicles (Figure 4C). Expression of COL4 (Figure 4D) was strikingly and significantly higher (Figure 4E) in adult ERβ-null focimatrix than in WT focimatrix, while expression of COL4 in other follicular compartments was similar in both genotypes, as observed in younger mice (Figure 4D).

### LAMININ

**Figure 5:**
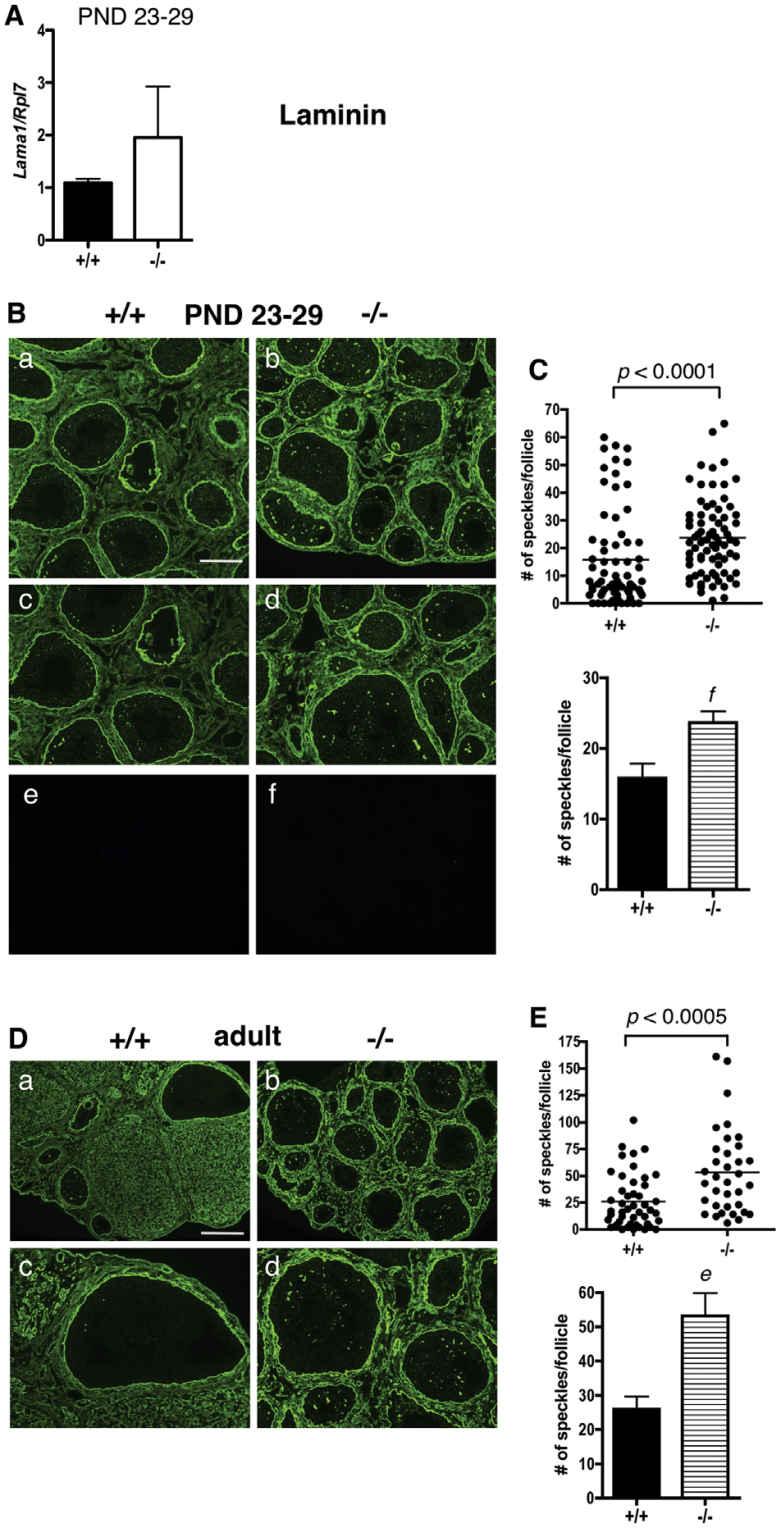
Laminin expression and localization in immature and adult ERβ-null and wildtype mouse ovaries. A. Granulosa cells were isolated and pooled from ovaries of untreated PND 23–29 wildtype (+/+) or ERβ-null (−/−) mice, and the levels of *Lama1* mRNA were determined by quantitative RT-PCR compared to an *Rpl7* control (± SEM of three independent experiments). B. Immunofluorescence with an anti-laminin antibody was used to detect laminin localization and expression in ovaries isolated from wildtype (+/+) and ERβ-null (−/−) mice at PND 23–29 (a–d; negative controls with secondary antibody only are shown in e and f) wildtype (+/+) and ERβ-null (−/−) mice. Two magnifications are shown. Scale bar = 200 µM for a–b, 100 µM for c–d. C. Focimatrix levels of laminin were quantified by counting the number of focimatrix speckles/follicle, and these values compared between genotypes by Receiver Operating Characteristic analysis (top panel) and a two-tailed, un-paired Student's t-test (± SEM, bottom panel). Each dot in the scatter plot (top panel) represents one follicle. f: *p*<0.005. D. Laminin expression in adult ERβ-null and wildtype mouse ovaries. Immunofluorescence with anti-laminin antibodies was used to detect laminin localization and expression in ovaries isolated from adult wildtype (+/+) and ERβ-null (−/−) mice. Two magnifications are shown. Scale bar = 200 µM for a–b, 100 µM for c–d. E. Expression of laminin in the focimatrix was quantified by counting the number of focimatrix speckles/follicle, and these values were compared between genotypes by Receiver Operating Characteristic analysis (top panel) and a two-tailed, un-paired Student's t-test (± SEM, bottom panel). Each dot in the scatter plot (bottom panel) represents one follicle. e: *p*<0.0005.

*Lama1* mRNA levels were similar in both ERβ-null and WT granulosa cells at PND 23–29 (Figure 5A). As previously reported [24], laminin was localized to the follicular basal lamina, the basal lamina of stromal blood vessels, the thecal matrix, focimatrix, and corpora lutea in both immature and adult mice (Figures 5B and 5D). At PND 23–29, ERβ-null follicles consistently possessed significantly higher numbers of focimatrix speckles per follicle than WT follicles (Figures 5B and 5C). Interestingly, laminin expression in the focimatrix of adult ERβ-null ovaries (Figure 5D) was again significantly higher than in WT focimatrix (Figure 5E).

## Discussion

In this study, we show that disrupted gene expression is observed in the ovaries of immature ERβ-null mice as early as PND 13, resulting in abnormal expression of ECM components in the ERβ-null ovary. We found that the mRNA levels of the ECM genes, *Col11a1* and *Nid2* were higher in granulosa cells isolated from ERβ-null PND 23–29 mice, or in whole ovaries isolated from PND 13 mice, than in age-matched WT controls. These elevated mRNA levels correlated with higher COL11A1 in the cytoplasm of granulosa cells and higher NID2 expression in the focimatrix of the immature ERβ-null ovary, at both PND 23–29 and PND 13. Interestingly, the elevated expression of COL11A1 and NID2 in ERβ-null follicles continued into adulthood. Finally, levels of the ubiquitous ECM proteins, collagen IV and laminin, were also higher in the adult ERβ-null ovary than in the WT ovary.

### An early role for ERβ in ovarian development

Our results showing that gene expression is dysregulated in ovaries of ERβ-null mice at PND 13 are consistent with studies suggesting that both the levels of ovarian ERβ and its ligand, E2, increase during a similar time-frame in post-natal ovarian development, and that E2 may act through ERβ at this time to regulate gene expression, and possibly follicle development. The presence of circulating E2 or its precursors has been established in neonatal rats [10] and mice [35], and androstenedione is detectable at PND 7 and increases dramatically at PND 15 [11]. ERβ protein is present and functional in the ovaries of PND 4–5 mice, but not in younger mice [13], [14], and ovarian ERβ protein levels increase with age [14], with the most abundant expression in granulosa cells. ERβ mRNA is detectable at PND 1 [14] or PND 4 [13] in the mouse ovary, with a dramatic increase occurring between PND 1 and PND 12 [14]. Evidence supporting a role for both E2 and ERβ in regulating primary and primordial follicle development in the mouse ovary has been suggested using various model systems [17], [19], [36], and our results showing disrupted gene expression in ERβ-null mice at PND 13 support a role in ovarian development in the immature mouse. Interestingly, during the period of human gestation when primordial follicles are formed, the fetal ovary expresses both the steroidogenic enzymes necessary for E2 production, and ERβ protein, suggesting that estrogen signaling may also regulate human primordial follicle formation [37]. While it may be possible that ERβ plays a role during prenatal ovarian development in the mouse, this is unlikely because ERβ mRNA is undetectable in the mouse ovary 26 days post-coitum [14] and only becomes detectable between PND 1 to PND 4 [13], [14]. Interestingly, although detectable at PND 8, we do not observe differences in gene expression by qPCR or protein levels by immunofluorescence in *Col11a1* or *Nid2* between ERβ-null and WT ovaries (data not shown) as we do at PND 13. One possible explanation for this lack of differential *Col11a1*/*Nid2* gene expression at PND 8 may be that ovarian ERβ levels are not high enough at PND 8 to detectably alter *Col11a1*/*Nid2* gene expression in WT mice, since there is a dramatic increase in ERβ mRNA between PND 1 and PND 12 in the mouse [14]. Thus it may not be until PND 13 that the lack of ERβ would result in significant differences in *Col11a1*/*Nid2* gene expression. On the other hand, there may be transcriptional coregulators required for ERβ-mediated transcription that are not present at PND 8 but are expressed at PND 13. Further experiments in WT and ERβ-null ovaries isolated from mice between PND 8 and PND 13 will be required to determine at which point during ovarian development ERβ activity is required for *Col11a1*/*Nid2* gene expression.

We have previously shown that ERβ-null granulosa cells isolated from PND 23–29 mice demonstrate an attenuated response to FSH, resulting in impaired *Lhcgr* and *Cyp19a1* expression, despite similar expression of FSH receptors [4], [5]. At least part of this attenuated response is due to reduced cAMP levels in response to FSH stimulation compared to WT granulosa cells [5]. Another important finding resulting from this previous study was that granulosa cells freshly-isolated from PND 23–29 ERβ-null ovaries produced significantly less cAMP than WT cells, even prior to stimulation by FSH. This reduced cAMP correlated with the elevated expression of phosphodiesterase 1c (PDE1C) in ERβ-null granulosa cells compared to WT cells (both isolated from untreated PND 23–29 mice) [5]. These results suggested that *prior* to PND 23, differences in granulosa cell gene expression between ERβ-null and WT mice are observed. Our current study supports and expands this observation, and provides strong evidence that the impaired ERβ-null granulosa cell response to FSH at PND 23–29 is also due to the dysregulation of perhaps numerous ERβ-dependent genes prior to PND 23 that are required to prepare a granulosa cell to fully respond to FSH at the onset of puberty.

Thus, we propose that ERβ, acting either through E2 or in a ligand-independent manner, regulates granulosa cell gene expression in follicles at various stages of growth: in the primordial, primary, or preantral follicle, and in response to FSH during the formation of a preovulatory follicle, as has previously been shown. While it is well established that E2 acting through ERβ is required to augment the granulosa cell response to FSH for the formation of a preovulatory follicle [4], [5], [38], [39], [40], [41], [42], fewer studies exist establishing a role for E2 in folliculogenesis, prior to the gonadotropin surge at puberty. Several reports indicate that E2 enhances or is required for the production of primary follicles [17], [19], although others suggest that E2 inhibits primordial follicle assembly [12], [36]. It has been reported that the number of primordial and primary follicles are similar in immature (PND 23) ERβ-null and WT mice, suggesting that ERβ is not required for the formation of primordial or primary follicles [18]. In contrast, adult female ERβ-null mice have elevated numbers of primordial follicles, and reduced numbers of primary follicles [18], suggesting that ERβ may participate in primordial follicle recruitment and/or maintenance. Further experiments will be required to determine the function of ERβ in the PND 13 ovary, and whether ERβ's loss at earlier stages truly impacts primary follicle formation and/or granulosa cell function.

### Disrupted Expression of ECM components in ERβ-null ovaries

This work is also novel in that we have characterized a significant elevation in multiple ECM proteins in immature and adult ERβ-null ovaries: a phenotype that has not previously been reported at either age. COL11A1 is expressed at very low levels in the WT immature ovary (Figure 1), but is robustly expressed in the cytoplasm of granulosa cells in the ERβ-null ovary at these ages. The localization of COL11A1 in the ovary of any species has, to our knowledge, not previously been reported, and in the mouse, *Col11a1* mRNA levels are highest in bone and cartilage [43]. In rat cartilage, COL11A1 is localized in the ECM between chondrocytes [44]; however, in human colon tissue, COL11A1 is localized to the cytoplasm (specifically, the Golgi apparatus) of goblet cells [45]. We also observed COL11A1 in the cytoplasm of granulosa cells, and the function of COL11A1 in granulosa cell cytoplasm certainly merits further study, as does the possibility that granulosa cells may secrete COL11A1 and contribute to granulosa cell-cell adhesion or migration. We also observe NID2 overexpression in the focimatrix in ERβ-null ovaries as early as PND 13 (Figure 2B), and NID2 remains elevated in the adult (Figure 2D). Similarly, laminin expression (Figure 5) was higher in the focimatrix of both PND 23–29 and adult ERβ-null ovaries compared to their WT counterparts, while COL4 was elevated in ERβ-null adult but not PND 23–29 ovaries (Figure 4). Our results are consistent with two previous reports in which global collagen levels were higher in adult ERβ-null ovaries than in WT ovaries, in either: a) both stromal and thecal layers [46], or b) in the stroma only [6]. Our work supports and expands these observations, indicating that not just collagen, but a number of other ECM proteins are aberrantly highly expressed in the adult ERβ-null follicle, and in addition, these elevated levels are observed in immature mice. The fact that NID2, laminin and COLIV expression was higher specifically in the focimatrix of ERβ-null ovaries, and not, for example, in the stroma, suggests that it is likely ERβ within granulosa cells regulating the expression of these genes (or other upstream genes required for their expression), since granulosa cells are the primary location of ERβ within the ovary, resulting in their secretion from the cell and localization to the extracellular region of granulosa cells. Further studies using *in situ* hybridization are needed determine which cells within the ovary produce these common ECM components.

### Regulation of *Nid2* and *Col11a1* by Estradiol and ERβ

There is evidence that E2 regulates ECM composition in the ovary and other tissues. For example, E2 regulates collagen turnover and ECM maintenance in the uterus and vagina of ovariectomized rats [47], and neonatal estrogen treatment disrupts the ECM composition of the rat prostate [48]. Abnormal ECM composition and structure is also observed in lungs of ERβ-null mice [49]. Within the context of our study, several hypotheses can be put forward to explain how the lack of ERβ results in increased expression of *Nid2* and *Col11a1* in the ERβ-null immature ovary. First, ERβ may directly repress the transcription of these genes either by binding EREs located proximal to or distant from the transcriptional start site, or by binding to other transcription factors, which themselves are bound to DNA (tethering). There is evidence that *Col11a1* and *Nid2* expression is regulated by E2 in other model systems. *Col11a1* mRNA is increased by E2 treatment of osteosarcoma cells expressing ERβ, but not ERα, indicating that not only is *Col11a1* regulated by E2 but that ERβ is selectively required for its regulation (Gene Expression Omnibus dataset GDS884) [29], although in this case E2 increases rather than decreases *Col11a1* expression, as would be predicted by the elevated *Col11a1* levels we observe in the absence of ERβ. Treatment of ovariectomized adult mice with E2 decreases uterine *Nid2* mRNA levels within six hours of treatment, consistent with a role for ERβ in repressing *Nid2* gene expression in the ovary [28]. It is also possible that ERβ indirectly decreases the transcription of *Nid2* and *Col11a1* by regulating the expression of other protein(s), such as transcription factors or transcriptional coregulators, or signaling molecules known to regulate folliculogenesis. In fact, in a whole ovary culture model in which PND 4 rat ovaries (which contain almost exclusively primordial follicles) were treated with Kit ligand [50], *Col11a1* expression was reduced, suggesting that Kitl signaling may be disrupted in ERβ-null neonatal ovaries. Lack of ERβ may also stabilize *Nid2* and *Col11a1* mRNA through regulation of a protein involved in RNA stability. Finally, it is possible that, ERβ may upregulate expression of a proteinase that degrades ECM proteins, resulting in the accumulation of NID2 and COL11a1, and perhaps laminin and COL4 as well, in the absence of ERβ. Further experiments are required to determine which of these potential mechanisms is responsible for the elevated expression of *Col11a1* and *Nid2*, and the other ECM proteins we observed elevated in ERβ-null ovaries.

### Potential impact of altered Expression of ECM components on ERβ-null ovaries

What impact the elevated levels of ECM protein in the cytoplasm (COL11A1) or in focimatrix (NID2, COLIV, laminin) of granulosa cells might have on folliculogenesis or function of the ERβ-null ovary is not clear. It is well established that dramatic changes in the ECM occur throughout folliculogenesis [24], [25], [27], and that the ECM carries out many functions within the ovary. Within the ovary and follicle, the ECM provides structural support, organizes and connects cells, and serves as a reservoir for signaling molecules that regulate follicle growth. The ECM also regulates establishment of the basement membrane, oocyte maturation, follicle atresia, steroidogenesis, and cell lineage [21], [26]. Further studies testing these specific functional endpoints in ERβ-null ovaries will help determine the potential impact of these overexpressed ECM proteins on ERβ-null ovary and granulosa cell function. The role of the focimatrix in granulosa cell and follicular function is less clear than that of the ECM, and very little is known regarding focimatrix function, although recent studies are beginning to address this question. Irving-Rodgers et al. have demonstrated that cholesterol side-chain cleavage cytochrome P450 (*Cyp11a1*) mRNA levels are highly and positively correlated with the expression of a number of focimatrix proteins in bovine ovaries, suggesting that the focimatrix participates in the selection of a dominant follicle [23], [31]. The same authors have also suggested that focimatrix may trigger the transition of an epithelial granulosa cell to a mesenchymal luteal cell by reducing the polarizing “cue” provided by the follicular basal lamina [30]. Thus, it is possible that the increased NID2, COLIV, and laminin expression we observe in the focimatrix of ERβ-null ovaries may impact the steroidogenic capacity of ERβ-null granulosa cells, and indeed, reduced E2 levels have been observed in cultured ERβ-null follicles [18]. Altered focimatrix composition may also affect ERβ-null granulosa cell luteinization, and this effect would be consistent with the dramatically reduced luteinization of ERβ-null granulosa cells in response to LH [4], [5], [37]. Also, given that focimatrix NID2 levels are lower in bovine partially dominant follicles than in fully dominant follicles or subordinate follicles, it is also possible that increased NID2 in focimatrix of ERβ-null ovaries may interfere with or alter follicle selection. Further experiments will be required to test these hypotheses.

A surprising finding was that NID1 mRNA is not elevated in immature ERβ-null granulosa cells, but that its protein expression is significantly higher in the focimatrix of immature ERβ-null follicles than WT follicles. Given this elevated NID1 expression observed in immature ERβ-null follicles, it was also surprising that NID1 focimatrix levels are similar in both genotypes in the adult mouse. One possible explanation for these findings is that ERβ may regulate export or secretion of focimatrix proteins such as NID1, and that attenuation of this activity might occur with age, resulting in similar NID1 protein levels in the adult ovaries of both genotypes. The ERβ-dependent regulation of focimatrix protein secretion may also explain the elevated focimatrix levels of COL4 and laminin observed in adult ERβ-null ovaries (and for laminin, also in immature ovaries), although COL4 and LAMA1 mRNA levels were similar in both genotypes in both immature (Figures 4 and 5) and adult (data not shown) ovaries. A final possibility to explain COL4 accumulation in the adult but not the immature focimatrix is that COL4 protein may begin to accumulate in the ERβ-null immature focimatrix, but differences between WT and ERβ-null may not be detectable until sufficient COL4 has accumulated in the adult to detect these differences. In total, these results suggest that not all focimatrix genes are regulated via the same transcriptional mechanisms, and that ERβ may differentially regulate focimatrix protein export, as differential mechanisms of export have previously been observed for individual ECM proteins [51], [52], [53]. Although co-regulated expression of *NID1*, *NID2*, and *COL4A1* mRNA has been previously observed in bovine follicles [23], species differences may also account for the lack of coordinated regulation we observe in the ERβ-null ovary.

In summary, we have shown for the first time that disrupted gene expression is observed in the ovaries of immature ERβ-null mice as early as PND 13, resulting in elevated expression of ECM proteins in the extracellular regions within the focimatrix or surrounding granulosa cells within the ERβ-null ovary. This increased expression is also observed in the adult ERβ-null ovary. These findings suggest that ERβ regulates gene expression in the ovary prior to puberty, and we speculate that dysregulation of ERβ-mediated gene expression in early postnatal life may disrupt folliculogenesis and/or contribute to the impaired response of immature ERβ-null granulosa cells to FSH [4], [5].

## Materials and Methods

### Mice

Experiments were performed in compliance with the guidelines set by the Canadian Council for Animal Care, and the policies and procedures approved by the University of Western Ontario Council on Animal Care (Protocol Number: 2007-042). The generation of ERβ-null mice has been described previously [8]. Mice were obtained from Taconic Farms Inc., NY. Immature ERβ-null (ERβ^−/−^) female mice were generated via breeding homozygous (ERβ^−/−^) males with heterozygous (ERβ^+/−^) females. Wildtype (WT) C57BL/6 females were generated via breeding WT males and females. WT females were used as controls in all experiments. All females were weaned at PND 21 and genotyped as previously described [8]. All studies were conducted with untreated animals (ie. no gonadotropin or any other treatment).

### Isolation of granulosa cells

Ovaries were removed from PND 23–29 mice and immediately transferred to a 100-mm cell culture dish containing 15 ml ice-cold M199 medium supplemented with 1 mg/ml BSA, 2.5 µg/ml Amphotericin B, and 50 µg/ml gentamicin (all reagents from Invitrogen, Carlsbad, CA). Ovaries were pooled according to genotype, and the granulosa cells from each were then expressed by manual puncture with 25-gauge needles followed by pressure applied with a sterile spatula. Follicular debris was removed manually and the granulosa cell suspension filtered through a 150-µm Nitex nylon membrane (Sefar America Inc., Depew, NY) mounted in Swinnex filters (Millipore, Billerica, MA). The granulosa cells were then pelleted by centrifugation at 250× g for 5 min at 4°C, followed by two washes in DMEM/F-12 medium containing 1% Penicillin/Streptomycin solution (Invitrogen, Catalog # 15070-063). The final cell pellet was frozen at 80°C.

### RNA isolation and quantitative RT-PCR

Frozen pellets of granulosa cells (PND 23–29 mice) or frozen whole ovaries (PND 13 mice) were solubilized in Trizol (Invitrogen, Carlsbad, CA) and RNA was isolated according to the manufacturer's protocol. RNA was further treated with DNaseI, then reverse-transcribed using Superscript II (Invitrogen). cDNA levels were detected using quantitative PCR with the ABI PRISM 7900 Sequence Detection System (Applied Biosystems, Foster City, CA) and Power Sybr Master Mix (Invitrogen). Primers were designed using the Applied Biosystems Primer Express Software version 2.0 (Table 1). Fold changes in gene expression were determined by quantitation of cDNA from target (ERβ-null) samples relative to a calibrator sample (WT). The gene for ribosomal protein L7 (*Rpl7*) was used as the endogenous control for normalization of initial RNA levels. Expression ratios were calculated according to the mathematical model described by Pfaffl [54], where ratio = (Etarget)^ΔCt(target)^/(Econtrol)^ΔCt(control)^ and E = efficiency of the primer set, calculated from the slope of a standard curve of log (ng of cDNA) vs. Ct value for a sample that contains the target according to the formula E = 10^−(1/slope)^ and ΔCt = Ct(vehicle)-Ct(treated sample).

**Table 1:** Primer sequences used for quantitative RT-PCR.

Gene	Accession #	Forward Primer	Reverse Primer
*Col11a1*	NM_007729.2	5′- AGTTGGTCTGCAGTGGCAATTTCG -3′	5′- AGATCCCAGATCCACCGTTTCGTT -3′
*Col4a1*	NM_009931.2	5′- CTCCAGGTCCCTACGATGTC -3′	5′- TCCAAAGGGTCCTGTCTCTC -3′
*Lama1*	NT_039658.1	5′- TCCGTGGATGGCGTCAA -3′	5′- TGTAGCGGGTCAAACACTCTGT -3′
*Nid1*	NM_010917.2	5′- CACAGGCAATGGCAGACAGT -3′	5′- CCCTTCACCTTGCCATTGA -3′
*Nid2*	NM_008695.2	5′- GTCTGTTTGGCTGGCTCTTTGCTT -3′	5′- TCCACGTCATGGACAAAGGTAGCA -3′
*Rpl7*	NM_011291	5′- AGCTGGCCTTTGTCATCAGAA -3′	5′- GACGAAGGAGCTGCAGAACCT -3′

### Immunofluorescence

Ovaries were dissected from PND13, PND 23–29, or two-month old adult WT and ERβ-null female mice and embedded in Cryomatrix (Fisher, Ottawa, ON). Using a cryostat, tissues were cut into 6 µm sections, mounted onto slides (Fisher) and stored at −20°C until use. Sections were fixed with 4% formaldehyde for 10 minutes, rinsed three times with phosphate-buffered saline (PBS), then permeabilized with 0.1% Triton X-100 for 15 minutes. Sections were again rinsed three times with PBS, blocked for 30 minutes with blocking solution (5% BSA in 0.1% Triton X-100), then rinsed three times with blocking solution. The tissue was then incubated for one hour with primary antibodies specific to each target, including rabbit polyclonal anti-nidogen 2 raised against a mouse epitope (1∶50, Santa Cruz Inc. sc-33143), rat monoclonal anti-nidogen 1 raised against a mouse epitope (1∶400, Abcam, Cambridge, MA, ab44944), rabbit polyclonal anti-collagen 11a1 raised against a human epitope (1∶200, Abcam ab64883), rabbit polyclonal anti-collagen IV raised against a mouse epitope (1∶500, Abcam ab19808), rabbit polyclonal anti-laminin raised against a mouse epitope (1∶200, Abcam ab11575), and rabbit polyclonal anti-calnexin raised against a dog epitope (1∶50, Enzo Life Sciences ADI-SPA-860). Sections were then rinsed three times in blocking solution and incubated in secondary antibody (FITC-conjugated goat anti-rabbit secondary antibody, 1∶250 Sigma F9887). The tissue was then washed twice in PBS followed by a 5 minute incubation in 4′,6-diamidino-2-phenylindole (1∶1000, Sigma), and slides were mounted with Vectashield (Vector Laboratories, Burlington, ON). Slides were stored at 4°C and visualized the following day with an Olympus Provis AX70 upright microscope. Images were captured using Image-Pro 6.2 Software.

### Statistical Analysis

Differences in average mRNA levels of *Nid2*, *Nid1*, *Col11a1*, and *Col4a1* between ERβ-null and WT granulosa cells as determined by qPCR were compared using an unpaired two-tailed Student's t-test. To estimate and quantify the amount of NID2, NID1, COL4, and laminin present in the focimatrix, the number of immunoreactive speckles per follicle in each follicle within the section was counted manually by an experimenter blinded to genotype. Atretric follicles were not included in the count. Speckles were counted in 21–78 follicles per genotype for each protein of interest from a minimum of three mice per genotype per protein. Larger aggregates of speckles were estimated based on a pre-determined minimum speckle size. The number of speckles/follicle was compared between ERβ-null and WT using two statistical tests. First, averages were compared using an unpaired, two-tailed Student's t-test. Second, differences were investigated using the more stringent criteria of Receiver Operating Characteristic (ROC) analysis, an analysis that tests for differences over the entirety of both distributions.

### Gene Ontology Analysis

The Database for Annotation, Visualization and Integrated Discovery 6.7 (DAVID 6.7) Functional Annotation tool [55], [56] was used to determine Gene Ontology Cellular Components [57] presented in Table S1 from a previously published dataset by Deroo et al [5]. All analyses were conducted with Maximum EASE Score/P value set to 0.05.

## Supporting Information

Figure S1 **Calnexin and COL11A1 localize to the cytoplasm of granulosa cells in ovaries of immature PND 23–29 mice.** Immunofluorescence with anti-calnexin (A) and anti-COL11A1 (B) antibodies were used to confirm the cytoplasmic localization of (A) calnexin in PND 23–29 wildtype mice, and (B) COL11A1 in PND 23–29 ERβ-null (−/−) mice (identical image to that in Figure 1C, section (f). (A): Scale bar = 100 µM; (B) Scale bar = 50 µM.
(TIFF)

Table S1 **Genes differentially regulated in ERβ^+/−^ granulosa cells relative to ERβ^−/−^ granulosa cells that were categorized as “Cellular component: Extracellular matrix proteins”.** Genes were categorized based on Gene Ontology annotations from the original gene expression analysis published by Deroo et al. [5] Genes are sorted by Fold Induction (Fold Induction = ERβ^−/−^/ERβ^+/−^). The Database for Annotation, Visualization and Integrated Discovery 6.7 (DAVID 6.7) Functional Annotation tool [55], [56] was used to determine Gene Ontology functions [57]. All analyses were conducted with Maximum EASE Score/P value set to 0.05.
(DOC)
