# Supplementary figures and images for: Expression of Extracellular Matrix Components Is Disrupted in the Immature and Adult Estrogen Receptor β-Null Mouse Ovary
Source: PLoS One. 2012 Jan 10;7(1):e29937. doi: 10.1371/journal.pone.0029937 (PMC3254630; doi:10.1371/journal.pone.0029937)

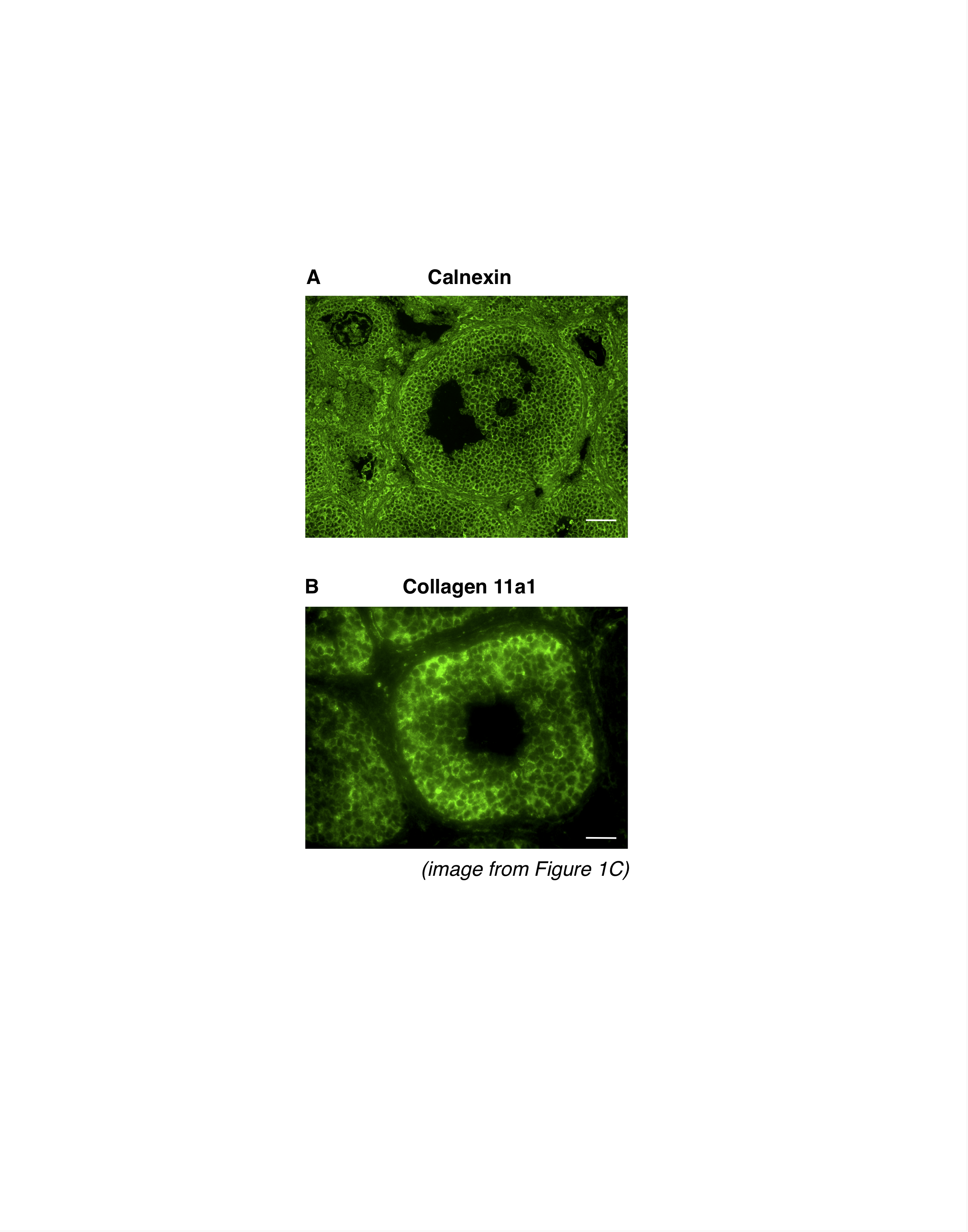

Supplement: Figure S1 — Calnexin and COL11A1 localize to the cytoplasm of granulosa cells in ovaries of immature PND 23–29 mice. Immunofluorescence with anti-calnexin (A) and anti-COL11A1 (B) antibodies were used to confirm the cytoplasmic localization of (A) calnexin in PND 23–29 wildtype mice, and (B) COL11A1 in PND 23–29 ERβ-null (−/−) mice (identical image to that in Figure 1C, section (f). (A): Scale bar = 100 µM; (B) Scale bar = 50 µM. (TIFF) [file pone.0029937.s001.tiff]
