# Supplementary material for: Expression of Extracellular Matrix Components Is Disrupted in the Immature and Adult Estrogen Receptor β-Null Mouse Ovary
Source: PLoS One. 2012 Jan 10;7(1):e29937. doi: 10.1371/journal.pone.0029937 (PMC3254630; doi:10.1371/journal.pone.0029937)
Supplement: Table S1 — Genes differentially regulated in ERβ+/− granulosa cells relative to ERβ−/− granulosa cells that were categorized as “Cellular component: Extracellular matrix proteins”. Genes were categorized based on Gene Ontology annotations from the original gene expression analysis published by Deroo et al. [5] Genes are sorted by Fold Induction (Fold Induction = ERβ−/−/ERβ+/−). The Database for Annotation, Visualization and Integrated Discovery 6.7 (DAVID 6.7) Functional Annotation tool [55], [56] was used to determine Gene Ontology functions [57]. All analyses were conducted with Maximum EASE Score/P value set to 0.05. (DOC) [file pone.0029937.s002.doc]

| Genbank ID | Gene Symbol | Gene Name | Fold | *P*-value |
| --- | --- | --- | --- | --- |
| NM_008695 | Nid2 | **nidogen 2** | 3.9 | 6.5E-42 |
| NM_178929 | Kazald1 | Kazal-type serine peptidase inhibitor domain 1 | 3.7 | 1.2E-40 |
| NM_007729 | Col11a1 | **collagen, type XI, alpha 1** | 2.3 | 1.4E-16 |
| NM_175506 | Adamts19 | a disintegrin-like and metallopeptidase (reprolysin type) with thrombospondin type 1 motif, 19 | 2.3 | 1.6E-07 |
| NM_016762 | Matn2 | matrilin 2 | 1.7 | 9.3E-10 |
| NM_008606 | Mmp11 | matrix metallopeptidase 11 | 1.6 | 0.00002 |
| NM_011775 | Zp2 | zona pellucida glycoprotein 2 | 1.6 | 9.5E-10 |
| NM_009369 | Tgfbi | transforming growth factor, beta induced | 1.6 | 0.00083 |
| AK078108 | Ptprz1 | protein tyrosine phosphatase, receptor type Z, polypeptide 1 | 1.5 | 0.00001 |
| NM_008482 | Lamb1 | laminin B1 subunit 1 | 1.4 | 4.2E-11 |
| NM_009368 | Tgfb3 | transforming growth factor, beta 3 | 1.4 | 3.1E-06 |
| NM_026439 | Ccdc80 | coiled-coil domain containing 80 | 1.4 | 0.00004 |
| NM_009929 | Col18a1 | collagen, type XVIII, alpha 1 | 1.4 | 9.5E-10 |
| NM_007833 | Dcn | decorin | 1.4 | 0.00002 |
| NM_175148 | N/A | RIKEN cDNA 2300002M23 gene | 1.3 | 0.00002 |
| NM_016696 | Gpc1 | glypican 1 | -1.2 | 0.00007 |
| AK003211 | N/A | RIKEN cDNA 1110001D15 gene | -1.3 | 0.00093 |
| NM_011261 | Reln | reelin | -1.4 | 0.00041 |
| NM_012050 | Omd | osteomodulin | -1.5 | 5.6E-09 |
| NM_010681 | Lama4 | laminin, alpha 4 | -1.9 | 0.00007 |
| NM_028266 | Col14a1 | collagen, type XVI, alpha 1 | -1.9 | 0.00001 |
| NM_145584 | Spon1 | spondin 1, (f-spondin) extracellular matrix protein | -2.5 | 6.1E-19 |
| NM_019919 | Ltgp1 | latent transforming growth factor beta binding protein 1 | -2.7 | 6.8E-20 |
| NM_016685 | Comp | cartilage oligomeric matrix protein | -15.7 | 7.2E-33 |
